# Measures used by stakeholders to mitigate Gender-Based Violence through the Ubuntu philosophy lens in South Africa

**DOI:** 10.3389/fsoc.2025.1587793

**Published:** 2025-05-26

**Authors:** Thingahangwi Cecilia Masutha, Angelina Maphula, Salaminah Moloko-Phiri, Molokedi Matsipane, Rodwell Gundo

**Affiliations:** ^1^Department of Advanced Nursing Science, Faculty of Health Sciences, University of Venda, Thohoyandou, South Africa; ^2^School of Nursing, North-West University, Mafikeng, South Africa; ^3^Department of Nursing, Faculty of Health Sciences, University of Pretoria, Pretoria, South Africa

**Keywords:** Gender-Based Violence, measures, mitigate, stakeholders, Ubuntu philosophy

## Abstract

Gender-Based violence (GBV) is a widespread problem in South Africa, impacting almost every aspect of life. This study aimed to explore measures different stakeholders use to mitigate Gender-Based Violence through the Ubuntu philosophy lens in South Africa. The study's objectives were (1) to explore how the Ubuntu philosophy can be applied to mitigate GBV in South Africa (2) to recommend culturally relevant, community-centered measures to mitigate GBV using Ubuntu principles and foster collective responsibility. The study was conducted in a selected university's boardroom in South Africa. A qualitative approach using explorative, descriptive, and contextual designs was adopted per the research objectives. The population comprised hospital nurses, Thuthuzela Care Center, social workers, psychologists, the South African Police Service (SAPS), the National Prosecuting Authority (NPA), and the Democratic Nursing Organization of South Africa (DENOSA). Eighteen participants were conveniently sampled and consented to participate. Semi-structured questions were used to achieve the objectives of the study. Two focus group discussions were used to collect data. The interviews lasted for 35–45 min and lasted for 1 day. Data was analyzed thematically using Tech's eight steps. Three themes with their sub-themes emerged: information giving, support needed, and Ubuntu principles to mitigate GBV, where reasons for women to remain in abusive relationships, culture and its influences on GBV, and GBV in the workplace were articulated. Mental health support, social development, and services available for the survivors were also deducted from the study as sub-themes. According to the study's findings, raising knowledge of the Ubuntu ideology and GBV may help lessen some types of GBV by promoting Ubuntu's values. This study recommends an interprofessional collaboration on curbing Gender-Based Violence using the Ubuntu philosophy.

## 1 Introduction

Gender-Based Violence (GBV) has been proven to be a problematic global phenomenon. It is characterized by gender inequality and its notable nature of human rights violations across societies (Anene and Njoku, 2020). According to the World Health Organization (2024), over a quarter of women aged 15–49 years who have been in a relationship have been subjected to physical and/or sexual violence at least once in their lifetime (since age 15). The prevalence estimates of Gender-Based Violence range from 20% in the Western Pacific, 22% in high-income countries and Europe, and 25% in the WHO Regions of the Americas to 33% in the WHO African region, 31% in the WHO Eastern Mediterranean Region, and 33% in the WHO South-East Asia region. In Malawi, one-third of Malawian women have experienced physical violence since age 15, one-fifth have suffered sexual violence, and 42% of women aged 20–24 were married before age 18 (World Bank Group, 2022; UN Women, 2025; Melnikas et al., 2021).

According to the Ghana Statistical Service (2023), the daily news of 04 December 2023 indicated that about 24.4 percent of women between 15–49 years' experience physical and/or sexual violence at least once in their lifetime. In addition, 19.2 percent of women aged 15–49 years' experience physical and/or sexual violence. GBV statistics in South Africa are alarming, being dubbed the “rape capital of the world,” with 10,818 rape cases reported in the first quarter of 2022 (Govender, 2023). South African Police Service Crime Stats of the third quarter of 2024/2025 on the serious and violent crime report overview of 03 December 2024. It has indicated that attempted murder increased by 2.2%, while contact sexual offenses saw a 1.2% rise in that quarter. These increases in more serious forms of violence and abuse suggest a need for continued attention and intervention in these areas. With all these statistics, GBV costs South Africa between R28.4 billion and R42.4 billion annually, which amounts to 0.9% to 1.3% of our GDP annually. Why is GBV so prevalent in South Africa? Is it because of the way males are brought up to exert power and control over vulnerable women? South Africa is riddled with patriarchal dominance, which is highly pervasive in every section and sector of society (Govender, 2023). That said, the country is grappling with GBV and femicide incidents, which some scholars regard as a dark stain on the realization of the sustainable development goals (SDG) target of eliminating all forms of violence in SSA.

Given this, there have been contingent measures crafted to curtail GBV and femicide in South Africa. The Presidential Summit on Gender-Based Violence and Femicide culminated in the adoption of the National Strategic Plan (NSP) on Gender-Based Violence and Femicide (Enaifoghe et al., 2021), which is in place in South Africa. The adopted NSP is a framework that reflects South Africa's national plan of action against Gender-Based Violence and serves as a policy guide for 2030 (The Presidency, 2020). There is also an Emergency Response Action Plan (The Presidency of South Africa, 2019) to combat Gender-Based Violence and Femicide (GBVF) in South Africa. In addition, the government has launched the Minimum Standards for Victims of Crime and Protection from Harassment Act (Republic of South Africa, 2011), which is related to the government's commitment to improving service delivery for victims of crime. It can be underscored that these measures have not mainstreamed the Ubuntu philosophy as an essential avenue in which GBV can be curtailed. However, there are very few studies about interventions to mitigate GBV, including the Ubuntu philosophy (principles and values), such as caring, empathy, compassion, and the collaboration of different stakeholders working together to achieve a common goal. Ubuntu is an African philosophy that emanates from a Nguni proverb, Umuntu Ngumuntu Ngabantu, which means “a person is a person because of or through others” (Fraser-Moleketi, 2009; Tutu, 2023).

This study aimed to explore measures different stakeholders use to mitigate Gender-Based Violence through the Ubuntu philosophy lens in South Africa. The study's objectives were (1) to explore how the Ubuntu philosophy can be applied to mitigate GBV in South Africa. (2) To recommend culturally relevant, community-centered measures to mitigate GBV using Ubuntu principles and fostering collective responsibility. This study's unique contribution is that its measures to mitigate GBV have been embedded with Ubuntu philosophy and the collaboration of stakeholders. This study contends that the Ubuntu philosophy is the backbone of African values, moral compass, and cultural consciousness. As such, it needs to be weaved with any measures poised to address African challenges, GBV and Femicide included. This study contributes to the novel debates on GBV and quests to find solutions to the conundrum by providing unique insights into the importance of Ubuntu philosophy in the fight against GBV.

## 2 Materials and methods

### 2.1 Research approach and designs

This study followed qualitative methods to answer the research question, describe life experiences, and give them meaning. Exploratory, descriptive, and contextual designs explored and described measures to mitigate Gender-Based Violence through the Ubuntu philosophy lens.

### 2.2 Sampling and sample size

The population of this study comprised of the following stakeholders, namely, one hospital nurse, one Thuthuzela Care Center, one social worker, one psychologist, one South African Police Service (SAPS), one member of the National Prosecuting Authority (NPA), and one member of the Democratic Nursing Organization of South Africa (DENOSA), one member of the Isa Mathivha Foundation, and one postgraduate nursing student. In addition, eight members of the selected four Universities who form part of the Ubuntu Model of nursing team participated in the study, making them 18. These participants were conveniently sampled.

### 2.3 Data collection

Data were collected through the focus group interviews. This technique was employed to allow for the in-depth exploration of the topic and generate rich quality data. The researchers had preconceived questions encapsulated in the interview guide, which allowed flexibility and openness in discussions. The main thread in the questions asked was about the measures that could be needed to mitigate Gender-Based Violence in South Africa. Then, the probing questions followed. Participants shared information about mitigating measures of Gender-Based Violence through the Ubuntu philosophy. Two focus group discussions were used: the first group, which was comprised of 10 participants, and the second group, which had eight participants. Participants consented to participate, and groups were divided based on expertise and field of work. The discussion guide comprised a set of questions that were used to facilitate discussions, and the responses were audio-recorded and later transcribed. The structure that was followed to facilitate the discussions included an introduction that also outlined the purpose and objective of the focus group, general questions in an open-ended format that allowed more engagement from participants, specific follow-up questions were also included and lastly, allowing participants to add anything in the end that they felt was important to be documented. The researcher led the sessions, and the assistant took notes and managed all recordings. Data was transcribed verbatim for analysis. The participants answered the questions until data saturation was achieved, prompting additional information. The interviews lasted between 35 and 40 min for each focus group for 1 day.

### 2.4 Data analysis

Data were analyzed using thematic analysis guided by Tesch's eight steps of the open coding method (Creswell and Creswell, 2017). (1) This allows researchers to understand all the transcriptions. Based on this, all transitions were read carefully, and essential points were jotted down. (2) One transcript was documented, and the researchers read it and asked about it. The researchers searched for the underlying meaning and documented the thoughts along the margin. (3) The researchers read and completed all the transcripts clustered together, listed similar topics, and formed these topics into columns. (4) The analyzed data were revisited, abbreviated topics as codes, documented next to the relevant text and checked if new categories and codes emerged. (5) The researchers decided on descriptive wording for the topics, which led to the development of categories. Data was reduced based on related topics, and lines were drawn to show the categories' interrelationships. (6) The final decision was made on abbreviations for each category, and alphabets were used for the codes. (7) A preliminary analysis was performed on the material belonging to the category. (8) The existing data was recorded to finalize the analysis.

### 2.5 Ethical considerations and trustworthiness

The selected university granted permission to conduct the study inside the institution. The Ubuntu project has an ethical clearance No: FHS/21/PH/17/131D. Participation was voluntary, and written consent was obtained from the participants. The following criteria for trustworthiness were adhered to: credibility, dependability, confirmability, transferability, and authenticity.

## 3 Findings of the study

### 3.1 Demographic information of participants

Participants were 18, comprising one female hospital nurse, one female Thuthuzela Care Center member, one female social worker, one female psychologist, one male South African Police officer, one female member of the National Prosecuting Authority (NPA), and one male member of DENOSA, one female member of the Isa Mathivha Foundation, and one female postgraduate nursing student. In addition, two males (a professor and a doctor) and six females (five professors and one doctor) members of the selected four universities who form part of the Ubuntu Model of nursing team participated in the study, making them 18.

### 3.2 Findings

Three themes with their sub-themes emerged: information giving, support needed, and Ubuntu principles to mitigate GBV, where reasons for women to remain in abusive relationships, culture and its influences on GBV, and GBV in the workplace were articulated. Mental health support, social development, and services available for the survivors were also deducted from the study as sub-themes. These themes and sub-themes are discussed in detail below.

#### 3.2.1 Theme 1: information giving

Information giving emerged as a first theme with the following sub-themes: reasons for women to remain in abusive relationships, culture and its influences on GBV, and GBV in the workplace. Each sub-theme is discussed below in detail.

##### 3.2.1.1 Sub-theme 1: reasons for women to remain in abusive relationships

One of the participants indicated that there are several reasons why women stay in abusive relationships. The participant also alluded that there are different reasons, and some of the reasons are fear of what people will say because they know her as a married woman, fear of not getting another husband, fear of disclosing that she is in an abusive relationship, financial dependence, hope that he will one day change.

The following quotes confirm the findings:

“*…Everyone knows that I'm a married woman; they end up being in that space because they don't want to be, you know, a laughingstock. If I decide to leave, what will they say? Sometimes, you know, it makes them feel unsafe to leave their homes and fear disclosing that I'm in an abusive relationship. So, they end up deciding to keep quiet. Another reason is financial dependence, which makes them feel inferior and lack self-esteem. Many women depend on their husbands for everything. …” FGD 2 P2*.

“*…Umm, so lack of financial independence, you know, limits their ability to live the abusive relationship as well because the minute you start thinking, I've got 4 kids, you know, how am I going to support them? Where am I supposed to go? I can't go back home because there's about the wife and the kids. So, they end up being stuck in that situation…” FGD 1 P3*.

“*…If I leave my home, whose husband will I take? I'd rather be stuck with my husband than leave him to go and disturb other people's marriages. And I always have that hope that he will one day change…” FGD 2 P4*.

“*One of my study findings found that patriarchy was the main reason why people stay in abusive relationships because, looking at the meaning of patriarchy, it promotes the supremacy of men, whereas women are just to be submissive; however, there's an author of the proverb who says even though ‘Lebitla la mosadi ke bohadi' has been seen to be promoting patriarchy. You know, it was not meant to be like that; it is only that people tend to use it to favor them or to favor their situations. However, most people continue to use this proverb...” FGD 1 P1*.

The above narrations suggest that most women stay in abusive relationships due to cultural norms and the fear of the stigma of divorce. Some are staying in an abusive relationship due to dependency issues as they are not working and they are not educated.

##### 3.2.1.2 Sub-theme 2: culture and its influences on GBV

Participants explained how cultural issues are viewed in the community. They spoke about the saying that once you are married, you cannot go back home and that it causes the oppression of women. Stigma is another issue during the interview, and they said they are worried about the stigma. Some indicated that people in the community believe that if your daughter is married, she should go and be a daughter-in-law. Another issue is that women don't talk back to their husbands. Another participant indicated that the women even sing a song that says that a wife should not ask about the husband's pay slip.

The following quotes confirm the findings:

“*… Even though there can be issues of violence in the family or the marriage, you know, the interpretation that was given through to people was that even though you can encounter issues of violence, you know, against women and children, one is not supposed to go back because they're told you belong there, you have been married…” FG2 P1*.

“*…You don't even have a room at home, and some women said that parents would just push them and say, OK, you can sleep over tonight, but tomorrow you must go. I don't want people to look at me and say, “Hey, she is bad…” FGD 2 P3*.

“*…It is even worse in our country. This GBV is worse when it is cultural because people will say, in my culture, women do not talk back to their husbands, and you don't ask anything about his pay slip…” FGD 1 P6*.

The above narrations suggest that most cases of GBV are influenced by cultural norms where men are given more power than women, and women don't have a say in their marriages. They are told that as a woman, you don't answer when your husband talks. Those are the issues that influence GBV actions.

##### 3.2.1.3 Sub-theme 3: GBV in the workplace

The participants stated that GBV in the workplace is a serious violation of human rights and an attack on the dignity and physical and psychological integrity of “staff members.” They further indicated that there are forms of GBV in the workplace, like bullying and shaming, physical and verbal abuse from work colleagues, supervisors, or managers, sexual harassment and unwanted sexual advances, sexual abuse, and violence. The following quotes confirm the findings:

“*…It is in the interest of all public and private employers to create a productive workplace and increase women's labor force participation by ensuring a safe environment for their workers…” FGD 2 P7*.

“*…Traditional gender roles and patriarchal beliefs contribute to the normalization of GBV in South Africa. These norms often perpetuate the notion that men have the right to exert control over women, including the use of violence to maintain dominance. When it appears normal, victims feel disarmed and desperate… FGD 1 P2*.

“*…With each organization having a policy on GBV, cases continue to rise. Many GBV incidents are not reported, which remains a significant challenge to address GBV in our workplace. Perpetrators continue to behave wrongly as they ‘won't be punished. The victims are blamed for the events they experienced. Victims withdraw from reporting their traumatic experiences. Perpetrators take advantage of the victim's silence…” FGD 1 P 4*.

The above narrations imply that most people are unaware of workplace bullying where harassment might happen, and they take it as a standard action because it is the boss or employer who is saying or doing it; his word is final.

#### 3.2.2 Theme 2- support needed

Three sub-themes emerged from theme two: mental health support, social development, SAPS, NPA, Care Centers, Lifeline, and Psychologist services.

##### 3.2.2.1 Sub-theme 1: mental health support

The findings show that mental health support is critical for victims of Gender-Based Violence because if mental health issues are not addressed, it has devastating effects not only for the survivor but also for those closely related who will interact with the survivor. It was emphasized that in an ideal world, when attending to a female survivor will assist the survivor in being at ease to be attended by a female professional to avoid evoking painful trauma for the survivor.

The following quotes confirm the findings:

“*…Mental health is critical; therefore, resolving trauma is of paramount importance given the aftermath if trauma is not treated, mothers will not be able to parent effectively if their trauma is unresolved…” FGD 1 P10*.

“*…Mental health interventions must also focus on the victims' emotional state with the application of intense therapy and counseling because of the negative impact that is brought by abuse, resulting in anxiety, post-traumatic stress disorder (PTSD), depression, etc...” FGD 1 P5*.

“*…They would be very anxious, and that would be emanating from the trauma because each time when we talk about GBV, we must know that people go through that trauma, and it would stay because unless it is resolved, it will stay, and it will always be torturing them. So, we find that the aftermath includes anxiety, and women or children will be depressed…” FGD 1 P7*.

“*…You know, there is intense counseling as that we have heard that, you know, it's not one size fits all because for someone who has gone through this experience, you will find that they have been hurt physically, maybe they see a scar in their face every day that reminds them of the physical abuse that went on…” FGD 2 P5*.

The above narrators depict that it is essential that the GBV survivor gets mental health support as the crime affects the survivor's mental health. Most of them suffer from different mental disorders due to trauma.

##### 3.2.2.2 Sub-theme 2: social development

Participants indicated that it is essential that all survivors understand the role of the social worker in combating GBV. The following quotations confirm the findings:

“*…Social workers play a crucial role in addressing gender-based violence because we do assessments, we provide counseling, provide therapy…” FGD 1 P5*.

“*…Additionally, we do awareness campaigns about GBV and other social life issues and training, and we link the survivors with relevant stakeholders…” FGD 1 P3*.

The above information indicates that social services are very important in mitigating GBV in our country as they are part of the multidisciplinary team, and they should be included in this fight against GBV.

##### 3.2.2.3 Sub-theme 3: SAPS, NPA, care centers, lifeline, psychologists

The current study also revealed that anti-violence centers are available to provide support directed toward victims of Gender-Based Violence. Such centers aim to empower and support victims by offering psychological counseling, legal counseling, and professional guidance. The following quotations confirm the findings:

“*…The police, psychologists, and various professionals are available to support victims, although there might be room to improve strategies employed…” FGD 2 P6*.

“*…But then we need more dialogues. We need more dialogue on the GBV issue collaboratively as stakeholders…” FGD 1 P4*.

“*…Lifeline is also another service that operates to combat the aftermath of gender-based violence among victims and assist them with the prevention of suicidal death as other victims resort to committing suicide after trauma…” FGD 1 P9*.

“*…In addition, we will increase awareness and support for people in crisis worldwide as well as online counseling....” FGD 1 P10*.

The above narrations indicate that awareness of the available services is very important to inform community members about where to get help during a crisis and after the attack.

#### 3.2.3 Theme 3: Ubuntu principles to mitigate GBV

The theme, Ubuntu principles to mitigate Gender-Based Violence, refers to pillars of our being that can help to mitigate Gender-Based Violence in our communities. Throughout the interviews, the participants identified and referred to the following principles: collectivism and solidarity, collaboration, solving problems to reach a consensus, and training Ubuntu ambassadors in schools. The following sub-themes emerged.

##### 3.2.3.1 Sub-theme 1: collectivism and solidarity

Collectivism and solidarity are about working together. The participants observed that the problem of Gender-Based Violence is so huge that the fight against it cannot be left to one person. The following quotations confirm the findings:

“*…There is a need for togetherness to address gender-based violence effectively...” FGD 2 P3*.

“*…Applying Ubuntu principles to combat the impact of gender-based violence requires collective efforts of different stakeholders, including the community members. Sometimes, community members protect the perpetrators when police officers look for them” FGD 1 P 1*.

“*…To attain quality outcomes in fighting GBV, effective collaboration within Provinces and countries between centers and other healthcare professionals is becoming increasingly important…” FGD 2 P6*.

The above narrations imply that people should work together in unity, supporting each other with the survivors of GBV instead of blaming them. Community working hand in hand with police officers, reporting cases of GBV.

##### 3.2.3.2 Sub-theme 2: collaboration

Related to collectivism and solidarity, collaboration refers to identifying and working with other stakeholders for a common cause. The participants noted that there are different partners and organizations whose focus is addressing Gender-Based Violence. The following quotations confirm the findings:

“*…Lack of collectivism and collaboration are to blame for the decline in Ubuntu and the increase of GBV cases in our country because everyone is trying to fight this issue alone in her corner. For example, SAPS is trying to fight this, and the community will protect the culprit. It is essential that we identify and work in unity as stakeholders…” FGD 1 P8*.

“*…Without collaboration, there would be a lack of professional relationships, a lack of teamwork, a decrease in the accomplishment of shared objectives and passions, a lack of benchmarking, a lack of knowledge and skill sharing across the country…” FGD 1 P5*.

According to the narrations above, GBV needs a collaboration strategy because all these stakeholders can work together to achieve a common goal: mitigating GBV.

##### 3.2.3.3 Sub-theme 3: solving problems to reach a consensus

The participants further observed that when addressing the problems related to Gender-Based Violence, people should aim to reach a consensus on what needs to be done. A consensus was defined as a widely accepted opinion. The following quotations confirm the findings:

“*…Collaboration allows us to resolve issues, bring fresh ideas, and work toward a common goal with one mind showing Ubuntu values; by doing that, we can win this battle of GBV…” FGD 2 P7*.

“*…This Ubuntu principle is relevant to the GBV mitigation because all stakeholders are facing the same challenge of fighting against GBV, and no one seems to win this, so it needs us as a country to be united and agree on the same strategies to mitigate GBV…” FGD 1 P2*.

The above findings and quotations imply that to win the GBV battle; stakeholders should come together and agree on ways to deal with the issue of GBV. One person can solve a small problem, but many people can solve a bigger problem.

##### 3.2.3.4 Sub-theme 4: training of Ubuntu ambassadors in schools

The participants reported that Gender-Based Violence can be addressed if the youth and learners learn and grow with Ubuntu. The following quotations confirm the findings:

“*…This can be achieved if learners are taught this philosophy and are requested to be ambassadors of the philosophy in their schools and communities…” FGD2 P6*.

“*… Maybe we should start training them in Ubuntu philosophy when they are still in primary school or even pre-school because they learn better when they are still very young, maybe between two and four, and make them a cohort which will be followed up, recorded until they become adults and see the impact of the training. Then we might have a better future parent with less rate of GBV…” FGD 1 P8*.

“*… To add to what she said, we can even encourage those who are being trained as ambassadors to make their mission to take two kids and hold them by their hands to teach them about Ubuntu philosophy. This can change our future country…” FGD 1 P9*.

The above narrations emphasize that training Ubuntu ambassadors in schools can be used as one of the strategies to mitigate GBV. However, it is a long-term strategy as it needs a follow-up on the cohort group that will be trained as ambassadors to check for the impact of the training.

## 4 Discussions

Information emerged as the first theme with the following subthemes: reasons for women to remain in abusive relationships, culture and its influences on GBV, and GBV in the workplace. Women remained trapped in abusive relationships because they did not want to be a laughingstock; therefore, they chose to stay in miserable relationships. This finding was reinforced by Ondicho (2013), who stated that women became trapped in violent relationships due to a lack of support and sympathy from family and friends, as well as geographical distance, which also contributed to the situation. Another study conducted in the United States by Pugh et al. (2021) found that women commonly stay in abusive relationships because they have hope for the future and a positive belief in the same relationship. Lack of financial independence was also stated as a reason for staying in the abusive relationship. This was supported by Antai et al. (2014), who reported that many abused women were concerned about the ability to support their children; therefore, they decided to stay. The findings are similar to those of Mulaudzi (2013), who observed that cultural ideas play an important role in communicating expectations in marriage or relationships to men and women. Therefore, women are to abide by the cultural rules and expectations of marriage/relationships without questions, which reflects the patriarchal system that oppresses women. On the other hand, Mtengwane and Khumalo (2020) from the City Press, the World Bank asserts that the characteristics related to female victimization include dependency on male partners and power dynamics in which the man feels superior. These factors are believed to expose women to unemployment and Gender-Based Violence.

In South Africa, Hoza et al. (2013) did a study highlighting that women are culturally socialized to participate in the cultural marriage that presents skewed power relations favorable to men. This socialization of women is typically accomplished through the employment of customs and proverbs such as “Lebitla la mosadi ke bogadi,” which translates to “A woman's grave is at the place of her in-laws,” as alluded to by the participants in this article. This is further supported by Mulaudzi (2013), who affirms that culturally, a woman is to remain married, live, and die at her husband's place even if the relationship is abusive to her and the children. For example, society expects women to live and die at their husband's place, even in abusive situations, as proverbs such as “Lebitla la mosadi ke bogadi” state. Similarly, Osiesi et al. (2023) argues that Nigerian society discourages women from expressing issues in public, regardless of the extent of violence in their marriages. Instead, women are encouraged to persevere for the sake of their children. Women are, therefore, expected to accept whatever happens in their marriages and to be utterly subservient to their husbands, which is the root cause of GBV. The findings suggested that women should have more productive and safe workplaces and increase their labor force participation. Once a safe employment atmosphere is established, women will feel safe and free to enter the labor market. This is reinforced by Kaphle et al. (2015), who conducted a study in Nepal and found that most women are not safe in the workplace because they are victims of Gender-Based Violence and are more likely to face other forms of violence. In support of the finding, Akter et al. (2024) revealed that women are frequently exploited in the workplace due to patriarchal institutions and their lower status in society. Furthermore, it is therefore essential that a safe workplace environment, policies, and programs are developed for women's overall wellbeing.

The findings of this study also show that traditional gender roles and patriarchal beliefs contribute to the control of GBV in South Africa, where societal norms often perpetuate the notion that men have the right over women and that they often use violence to maintain dominance. It is further revealed that even though organizations have a policy on GBV, the number of cases keeps increasing. This is confirmed by Oyelana and Ngcamu (2023) of Labor Laws News South Africa, who report that according to the 2022/2023 quarter one crime statistics, 855 women were killed in South Africa as the workplace has expanded beyond what is formally known as the “workplace” into employees' homes. As a result, the likelihood of employees becoming victims of GBV has grown, even while at home, and perpetrators often go unpunished. Furthermore, Ssanyu et al. (2022), who conducted a study in Uganda, support the findings that many GBV incidents are not reported, which creates a challenge in controlling the scourge. The same author asserts that when Sexual Gender-Based Violence (SGBV) is reported, survivors have access to medical, psychological, and legal services, and the prevalence of the violence can be accurately estimated.

The findings revealed that women affected by GBV should be assisted compassionately following the painful encounter so that they can carry out their parental responsibilities. Based on the study's findings on interventions for improving mental health outcomes of GBV-affected women, St. John and Walmsley (2021) argue that barriers to care should be removed, interventions should be successful, and long-term outcomes should be improved. Furthermore, affected women should be treated fairly and with respect, and there should be awareness campaigns to improve the mental health of abused women. This demonstrates the importance of applying Ubuntu principles to promote the mental health of women who have experienced GBV. The study's findings revealed that police officers should be available to assist victims of GBV. According to Simelane et al. (2023), members of the South African Police Service (SAPS) must be able to adapt to the changing demands since GBVF places a significant load on the police and other public agencies. To address the incidence of GBVF, an urgent, coordinated, and uncompromising emphasis on the entire system is required to aid victims (Moganedi and Mohlatlole, 2024). Recommend that all survivors of GBV who attend a victim-friendly room in a police station be assessed by a social worker for psychosocial support. Furthermore, the authors assert that the Department of Social Development has obtained more vacant buildings in South Africa, increasing the availability of facilities for survivors to receive psychosocial support. The psychosocial support services include shelters and the Khuseleka One Stop Center. These institutions serve as a one-stop shop for victims and survivors of GBV, providing integrated services under one roof. The Departments of Social Development, Health, Justice, the National Prosecuting Authority, and SAPS provide the abovementioned services.

## 5 Conclusion

This study's unique contribution is that its measures to mitigate GBV have been embedded with Ubuntu's philosophy and the collaboration of stakeholders. This study contends that the Ubuntu philosophy is the backbone of African values, moral compass, and cultural consciousness. As such, it needs to be weaved with any measures poised to address African challenges, GBV and Femicide included. This study contributes to the novel debates on GBV and quests to find solutions to the conundrum by providing unique insights into the importance of Ubuntu philosophy in the fight against GBV. The limitation of the study is that the data was collected in 1 day, which might have been limited due to time constraints. The study recommends conducting further studies using a mixed-methods approach to assess the long-term impact and sustainability of the measures explored.

## Data Availability

The raw data supporting the conclusions of this article will be made available by the authors, without undue reservation.
